# “Girls Have More Challenges; They Need to Be Locked Up”: A Qualitative Study of Gender Norms and the Sexuality of Young Adolescents in Uganda

**DOI:** 10.3390/ijerph15020193

**Published:** 2018-01-24

**Authors:** Anna B. Ninsiima, Els Leye, Kristien Michielsen, Elizabeth Kemigisha, Viola N. Nyakato, Gily Coene

**Affiliations:** 1 RHEA Centre for Gender, Diversity and Intersectionality, Vrije Universiteit Brussel (VUB), 1050 Brussels, Belgium; gily.coene@vub.ac.be; 2 School of Women and Gender Studies, Makerere University, 7062 Kampala, Uganda; 3 International Centre for Reproductive Health, Ghent University, 10 UZ-P114, 9000 Ghent, Belgium; els.leye@ugent.be (E.L.); Kristien.michielsen@ugent.be (K.M.); ekemigisha@must.ac.ug (E.K.); 4 Faculty of Interdisciplinary Studies, Mbarara University of Science and Technology, P.O. Box 1410 Mbarara, Uganda; vnyakato@must.ac.ug

**Keywords:** gender norms, early adolescence, sexual health, agency, Uganda

## Abstract

Unequal power and gender norms expose adolescent girls to higher risks of HIV, early marriages, pregnancies and coerced sex. In Uganda, almost half of the girls below the age of 18 are already married or pregnant, which poses a danger to the lives of young girls. This study explores the social construction of gender norms from early childhood, and how it influences adolescents’ agency. Contrary to the mainstream theory of agency, which focuses on the ability to make informed choices, adolescents’ agency appears constrained by context-specific obstacles. This study adopted qualitative research approaches involving 132 participants. Of these, 44 were in-depth interviews and 11 were focus group discussions, parcelled out into separate groups of adolescents (12–14 years), teachers, and parents (*n* = 88), in Western Uganda. Data were analysed manually using open and axial codes, and conclusions were inductive. Results show that gender norms are established early in life, and have a very substantial impact on the agency of young adolescents. There were stereotypical gender norms depicting boys as sexually active and girls as restrained; girls’ movements were restricted; their sexual agency constrained; and prevention of pregnancy was perceived as a girl’s responsibility. Programs targeting behavioural change need to begin early in the lives of young children. They should target teachers and parents about the values of gender equality and strengthen the legal system to create an enabling environment to address the health and wellbeing of adolescents.

## 1. Introduction

Gender and unequal power relations within which sexual identities, beliefs and values are built [1] play a key role in the sexual wellbeing of adolescents [2,3]. Numerous studies indicate that unequal power and gender norms expose girls and women to the risk of HIV, early marriages, pregnancies and sexual violence [2,3,4,5]. It should be noted that decisions taken by girls and boys may not only depend on the knowledge they have, but may be influenced by contextual factors like societal values, and financial deprivation [5,6]. Social contexts and interpersonal relationships considerably contribute to the processes that shape adolescents’ sexuality [5]. The socialisation in childhood shapes how girls and boys live out their lives as women and men—not only in the reproductive arena, but in the social and economic realm as well [6]. According to Bandura [7], sex role behaviour is promoted by active parental training in sex appropriate interests and expectations. This process is referred to as role modelling, imitation, or observational learning [5]. This takes place ‘before young children get an opportunity to observe and discriminate the sexual appropriateness response patterns displayed by adult males and females’ [7]. Humans learn prevalent, accepted, or desired behaviours referred to as social norms [5] by observing the behaviours of valued social referents, such as parents, teachers, peers, neighbours and the media [7]. According to Bandura, identifying the sources of emulated behaviour can quite often be problematic given that children are exposed to multiple models [7]. Adolescents might be motivated to conform to behavioural norms because it attracts certain rewards (like acceptance) and contrary to that may attract punishments, such as social rejection, or decrease in social status [5].

The modelling is a continuous process. However, explicitly during adolescence, the world expands for boys by allowing them to enjoy privileges reserved for men; while girls endure new restrictions earmarked for women [6]. The norms that dictate girls to behave like girls [8], and conceptions of female sexuality as passive, devoid of desire, and subordinate to male needs or desires [9] make it difficult for women to negotiate safe sex [10].

Commitment to deal with gender inequalities by embedding gender equality in comprehensive sexuality programmes is a core criterion [11]. The Convention on the Elimination of all Forms of Discrimination Against Women (CEDAW, 1979), is an international human rights convention for the advancement of women and gender equality [12]. The 1994 International Conference on Population and Development (ICPD) Programme of Action focused on promoting human rights, advancing gender equality and improving sexual and reproductive health [13]. Integrating gender is not only a matter of human rights [13] but according to Tolman [9], any intervention that ignores this may be counterproductive or even dangerous.

Some programmes to adolescent health take the individualistic approach where knowledge, and attitudes have become important units of analysis in sexuality programmes [14]. While such approaches may exclusively focus on reducing adolescent risky sexual behaviours, they may fail to explain the multifaceted and multi-determined social processes [2] that facilitate or block such risks. As some studies have indicated [15], there is need to move beyond individualistic approaches towards approaches that address the socio-economic structural dynamics which affect individual risks [14]. Burns [16] indicates that even if girls had the information and skills necessary to have healthy sexual relationships, power imbalances in gender relationships render them powerless in the face of masculine sexual freedom. The dependency syndrome, in particular, the flow of money and gifts being predominantly from males to females renders girls more powerless in African countries [4].

In this paper we examine the degree to which sexual gender norms in Western Uganda are established early in the lives of adolescents; how the norms are perceived; and the effect this has on adolescents’ sexual agency, defined as the ability of Uganda’s 12–14-year-old adolescents to make purposeful choices and negotiate safe sexual relations in the context of unequal power relations. Authors such as Amartya Sen conceptualize agency as the ability to make purposeful and informed choices [17]. However, this notion has been challenged on the premise that this is rather a Western conceptualization of agency, that relies on individualistic notions of choice and autonomy [18]. For example, Dutta [19] and Kabeer [18] argue that individuals exercise their agency within existing social conventions, values, sanctions and relationships. Social and cultural environments can be constraining or enabling of individual agency [20]. Especially where community structures and cultural traditions are still strong and valued, individualistic and autonomous agency may be constrained by social norms [5].

Few studies have been conducted in Uganda and Sub-Saharan Africa on adolescent sexual agency. For example, Bell argues that young women in Uganda are agentic, using sexual relationships to improve their own situation by extracting money or gifts for sex from men [21]. Michielsen finds older adolescent girls in Rwanda being active agents in transactional sex [22]. Michielsen’s finding is similar to Nyanzi’s [23] whose participants gave various reasons for transactional sex by arguing that ‘nothing is for free’. While Nyanzi interprets this to be agency, she acknowledges the fact that relative poverty has a role to play given that parents are often unable to provide adequately for adolescent girls. It is therefore important to understand that the agency which is considered by Bell, Michielsen and Nyanzi could be very constrained by socio-economic conditions; and thus can only be understood in relative terms and within its relationship with the structures that girls operate in [19]. It should also be noted that the studies above have studied older adolescents, majorly between 15–20 years of age. Other studies, like Muhanguzi [3] and Iyer et al. [24], discuss the norms among older adolescents in central Uganda. It was important to understand the level at which adolescents internalize gender norms to inform programming.

Accordingly, this study brings to light new knowledge on the gendered sexual norms among young adolescents in South Western Uganda—even though some of the findings merely corroborate the general theoretical claims and empirical observations made elsewhere in the developing world on gendered power relations among older adolescents.

### Young Adolescents and Sexuality in the Ugandan Context

Uganda has about 46 percent of girls below 18 years already married, and 20–24 percent married or pregnant before they are 15 years [25]. Early marriages and pregnancies account for 20 percent of maternal deaths and those who survive suffer lasting complications like fistula and disability [26]. While sexuality education has been identified as one of the remedies to such risky behaviours [27], in Uganda and many African countries, sex and sexuality are a private matter and openly discussing sexuality is regarded as a taboo [23,24,25,26]. Sex is legitimatised in marriage [24] and virginity of girls before marriage is a highly expected cultural norm. Highly gendered sexuality education (SE) was carried out through indigenous institutions of the extended family and the community [16,17,18,19,20,21,22,23,24,25,26,27,28,29] where paternal aunties (known as *Senga* in Central Uganda and *Shwenkazi* in Western Uganda) had an important role in preparing girls for womanhood and marriage. Emphasis was, and still is, on the control of girls’ sexuality. Ironically, virginity was, and continues to be, highly valued for girls, but not for boys—even though the emphasis placed on the girl’s virginity at the time of marriage appears to be changing [30].

The patriarchal tendencies in Uganda are undeniable and polygamy remains a strong cultural norm in some communities [31]. While polygamy is not acceptable according to the dominant Christian values in Uganda, it is still culturally accepted and has been revitalised as “informal polygamy”, in which men have relationships with multiple women under the term “modern polygamy” [32]. Men’s control of resources within households persists, with men considered as the heads of the household, main decision-makers and in control of women’s sexuality and movements. However, according to Bantebya et al. [32] ideals of marriage are growing more fragile with women taking on new roles, for example, the participation of women in public affairs may leave many men feeling disempowered [32]. Challenging traditional masculinities can contribute to more domestic violence as a response to the cultural shock and unwillingness by some males to adapt to changing roles [32].

Uganda has numerous laws that have been enacted to protect the rights of children and fight gender inequality. These include but are not limited to the Constitution of Uganda 1995. For example, Article 32 (2) provides that the “Laws, cultures, customs and traditions which are against the dignity, welfare or interest of women or any other marginalized group … or which undermines their status, are prohibited by this Constitution”; The Children’s Act (amended in 2016)—under Chapter 59 puts into effect the Constitutional provisions on children and emphasises the protection of the child by upholding their rights; The Domestic Violence Act, 2010, is another gender-friendly piece of legislation in Uganda. The Ugandan defilement law within the Penal Code Act (originally from 1950 which Uganda inherited from British colonial rule), has gone through some changes, whereby the age of sexual consent was increased in 1990 from fourteen to eighteen years. The 1990 edition of the law is very clear in the sense that it is not only illegal for a man to have sexual intercourse with a girl regardless of consent under the age of eighteen years, but also punishable by death [33,34]. As discussed by Parikh [35], the defilement law was defended by women’s rights activists and intended to address the social and health inequalities affecting young women and girls. However, the law was controversial and perceived as undermining men’s traditional authority and access to younger women for marriage or sexual relations. It was also criticized on the grounds that it was an encroachment by the state on culturally private matters that should be handled by the community [35]. Although the law intended to prosecute “sugar daddies”, Parikh found in her ethnographic study that it rather enforced patriarchal control and class hierarchies.

In 2007, the law was again amended and now includes women offenders and male victims. A person who performs a sexual act with another person who is below the age of 18 years commits an offence punished by a maximum period of life imprisonment. Under aggravating circumstances, the death penalty is sentenced. The law states that where the offender in the case of defilement is below 12 years of age the Children’s Act is applied. The Children’s Act states that a child above the age of 12 can be arrested and charged if he or she is suspected to have committed an offence, but a child below the age of 12 years cannot be charged with a criminal offence [36].

## 2. Methods

### 2.1. Study Design

The study aimed at exploring the gender-related sexuality norms among young adolescents in Uganda. The study used qualitative methods, in particular in-depth interviews (IDIs) and focus group discussions (FGDs). This research is part of a bigger project implementing sexuality education among young adolescents in primary schools in Mbarara district in Western Uganda. The total population of Mbarara district is roughly 472,629 with a total area of 1846 km^2^ [37]. The district is occupied largely by the Banyankole ethnic group. The literacy rate for persons above 18 years is about 82%, 87% for persons between 10–17 years and 42% for persons aged 60 and above. Data show that 46.7% of persons above age 10 and 62% above 18 years of age are married in Mbarara District. Agriculture (livestock or crop growing) is the major economic activity engaging 70% of the households [37].

Field work was done between July and August 2016 in Mbarara district with a total of 132 participants. Of these, 44 were in-depth interviews and 11 were focus group discussions, 44 in-depth interviews (IDIs) were done with school adolescent girls (*n* = 12) and, boys (*n* = 12) aged 12–14, teachers (*n* = 10) and parents (*n* = 10). 88 individuals took part in 11 focus group discussions (FGDs) performed in three rural, one semi-urban, and two urban schools. Four FGDs involved school adolescents (*n* = 32), and four FGDs with teachers (*n* = 32) and three FGDs with parents (*n* = 24) were conducted. Purposeful samplings were applied to select the six schools and all the participants. Teachers were selected based on their expertise or involvement in sexuality education. They mainly included senior women, senior men, science teachers, religious education teachers or deputy head teachers. Parents were chairpersons of boards in the school or parent-teacher associations and parents of the children that we interviewed. 

The rural–urban divide was purposively designed to account for any differences in sexual behaviours of the pupils, the level of knowledge, the involvement of parents, and the gender norms prevailing among adolescents in rural and urban settings. The parents in the rural setting were mainly small-scale farmers while parents in the urban setting were mainly involved in businesses or formal sector jobs. 

For the data collection we used interview guides. The guide for pupils mainly focused on their gender roles, gender and the source of their knowledge and prevailing gender norms regarding sexuality. The guide for teachers mainly focused on their knowledge and attitude towards gender, the prevailing gender norms in the schools and the role of parents. The guide for parents focused on the level of communication with their children, the knowledge they impart in their children (how gendered) and whether they approved sexuality education in schools.

### 2.2. Data Analysis

Interviews and FGDs were recorded, transcribed, (some) translated from local language (Runyankole) to English. Data was read and re-read and then open coded. In open coding, data were examined on differences and similarities [38] from which concepts were derived. Concepts were based on words or phrases that were used in open coding. Phrases included “like myself”, “do not like myself”, “menstruation”, “aspirations”, “domestic chores”, “girls leave school when they get pregnant”, and “money for sex”. Concepts like self-esteem, assertiveness, strong and weaker sex, gender roles, initiation of romantic relationships, among others were identified. We then preceded with axial codes where similar codes from different participants were grouped together, themes created and categories formed from which sections in this paper were derived. Data were identified to find the role played by the context in influencing participants perceptions [38], for example to identify the differences between rural/urban context and male/female perceptions.

### 2.3. Ethical Clearance

The study obtained approval from the Institutional Research Ethics Committee of Mbarara University (reference MUIRC 1/7) and the Uganda National Council of Science and Technology (reference SS 4045). At the beginning of each interview and focus group, written and verbal informed consent of teachers and parents was sought. Informed written assent of adolescents was obtained plus written informed consent from either the parents or the head teachers of the schools that adolescents attended. We sought consent on whether we could tape record their voices. We also obtained approval from school head teachers of the participating schools and the district administration.

## 3. Results

### 3.1. Sexually Active Boys and Restrained Girls

Field findings from Mbarara reveal that masculine behaviour is encouraged among boys and passivity and meekness promoted among girls with particular regard to their sexuality. In the interviews, boys easily talked about their sexual relations. Boys that had girlfriends and those that had engaged in sexual activity were more open about it, compared to the girls. Boys also expressed that they enjoyed larger freedom to explore sexual relations and seemed to be proud with their sexual activity. In one of the rural FGD with eight boys, all boys said they were sexually active except one;

When did you start to sleep with your girlfriend?

Boy 1: I was 11 years.

Boy 2: I was in primary 4 (12 years).

Boy 3: As for me, I’m 14 years and still don’t have a girlfriend. I need to first grow up and be responsible for my actions.

Boy 1 (interrogating and ridiculing Boy 3): What are you waiting for? Are you a tree? Do you think you will be source of timber? Laughter from all…

Interviewer: Does anyone of you have more than one girlfriend?

Boy 1: Yes, I have two. One in this school and another in a different school. Just in case one decides to leave me or if she behaves badly, then I have an alternative. Besides, I need to taste different girls.

Interviewer: But supposing she finds out that you have another?

Boy 4: Ohhh if she sees you that’s the end. You can touch them when she is not around.

Interviewer: Are these the girlfriends you intend to marry?

Boy 4: Haaaa, normally you stay with her like for two or three terms and if by that time you have slept with her like three times, you will have gotten tired of her and you leave her. Or she would have developed bad manners of going after other boys.

The quote illustrates how the masculine power of ‘*tasting girls*’ and control of female sexuality has already been formed by the age of 14 years. Boys state that they can have two or three girlfriends but that it is abnormal for a girl to do the same. However, it should not go without mention that girls are not merely passive. Their contestations regarding multiple partners by their boyfriends are an indication that girls are active participants.

However, the social norm that girls need to behave like girls was manifested in their refusal to open up about their own sexual relations during the IDIs or FGDs and to talk solely about other girls in their age group or in the same classes. Societal expectations by elders that girls should stay virgins until marriage, make it difficult for the girls to share such experiences. Even when she is not a virgin, she is supposed to pretend that she is, even among fellow girls:

Interviewer: Do some of your friends have boy friends?

Girl: Yes, some have, but they cannot tell you even if they had (IDI Girl Rural).

One of the informants revealed that she has a boyfriend who gives her gifts but indicated that she does not have sexual intercourse with him:

Girl: I cannot let my friend know my secret? Noooooo!! There is no one you can trust under the sun now. So I keep it to myself. I tell my blanket (IDI Girl rural).

Other informants indicated that when a girl is engaged in sexual relations, even if she is forced, it is still her fault. Furthermore, girls have been taught to be responsible and getting pregnant is solely seen as their responsibility:

*I don’t know whether there are unwanted pregnancies because one goes there willingly. But I hear people calling it unwanted pregnancies*.

(Head teacher semi urban)

*Sometimes girls dress inappropriately and cause men to rape them. Others cause it to happen because they eat men’s money and accept to meet somewhere not knowing that they may be raped*.

(FGD, Girl Rural)

*When you get used, spoilt and get pregnant, you stop going to school and start suffering. Your friends start talking about you, you lose respect and you are labelled with ugly names. And if your parents chase you away you become homeless and may become a housemaid… Nothing happens to the boy. They continue studying, unless they refuse to continue on their own. But getting a girl pregnant does not make them drop out of school*.

(IDI Girl rural)

*Of course men do not face as many challenges as women. When a girl gets pregnant, that’s her end. For me I was told by my mother that girls who get pregnant before marriage are thrown into river Kisiizi. I never wanted a man to touch me because I thought if a man touches me I get pregnant*.

(Female Parent, urban)

While there is a law against defilement in Uganda, its enforcement has been ineffective or selective. The men or boys that impregnate girls may either deny paternity, escape, or get into police cells for a few days, only to be released.

A boy who participated in an FGD reported as follows:

*It is better to impregnate someone’s daughter than to steal. Moreover, our parents find a boy who impregnates girl more socially acceptable than a girl who gets pregnant*.

(Boy FGD rural)

A teacher who participated in our focus group discussion rhetorically asked the question:

Have you ever heard a defilement case taken serious? She hastened to answer as follows: “No! The laws in Uganda do not work”.

A male parent from rural Uganda—reported:

“I took a boy who impregnated my 16 years old daughter to police; he was released in a few days. We get discouraged by the police.”

A female parent from Mbarara municipality faulted government for its incompetence. She argued:

“The government is negligent. A young man I know raped an adolescent girl of my daughter’s age. Police imprisoned him, but he was released after five days once the parents pleaded with the police. By failing to be tough on offenders, the government itself encourages criminal behaviours. The government should do more. Arrest the criminals and imprison them for a long time.”

Girls are socially expected to take care of their own sexuality but also take responsibility for their male sexual oppressors. It is perceived to be a girl’s duty to safeguard herself from the uncontrolled feelings of boys and men. Some girls indicated that when boys write love letters to them or harass them, they dread telling their parents because some parents blame girls for the behaviour of the boys:

Interviewer: Do you readily share information with your parents if a boy writes you a love letter or makes advances for a sexual relationship?

Girl: *No! I received a love letter from a boy. I feared to tell my mother about the letter but I told a teacher. I discerned that my mother might think that I brought the problem upon myself yet that was not the case. There is also a boy in our neighbourhood who touches me inappropriately when my parents are away. I wanted to talk to my mother but I still fear her because she might think I am drawing the advances myself* (IDI Girl semi-urban).

*Some parents can respond harshly by telling you that perhaps you want to sleep with a man if you start sharing information about sexual advances or ask questions about sexuality* (IDI Girl Rural).

*My mother is tough. She is likely to say that the boy would not have approached me if I had not shown interest by familiarizing with boys for example, by smiling in a seductive way* (IDI Girl semi-urban).

Parents and teachers believe that girls’ freedoms need to be restricted because girls have more to lose in a relationship than boys. A senior woman (urban) succinctly stated:

“Girls have more challenges in this modern world. With this exposure, girls need to be locked up.”

A male rural-based parent seemed to agree. For him:

“Girls are like ground nuts, which are a delicacy in Uganda. Everyone wants to pick and eat them. Parents to girl children must take extra effort to control and discipline them.”

Another rural-based parent from a cattle-rearing community, likened girls to milk—another precious but fragile product. For him:

“Girls are like milk-it keeps on attracting house flies. As a parent, you have to safeguard your daughters more than you do to your sons. For the boy-child you can send him to school by himself, but the girl child needs to be escorted…”

A rural-based girl reported as follows:

*“Parents restrict us from visiting our friends. They seem to think that a girl might have a ‘deal’ to meet a boy and engage in premarital sex. But boys do not get spoilt like girls so they allow them to go. Even if a boy delays to come, he still comes back home without much trouble. Besides girls have many responsibilities, there is always something that you are expected to do at home*.”

(IDI Girl Rural)

Teachers and parents believed that only girls need sexuality education because they have higher risks than boys. While schools have senior women and senior men who are both responsible for counselling adolescents about maturation, it was only the senior women who were actively involved in sexuality education in all the schools that we visited. The senior women met girls at least 2–3 times a term (three months) while boys could spend a term without meeting the senior man:

*As for these old boys, I meet them whenever there is a trigger or a signal that something is wrong. We don’t normally give them too much attention as girls because in primary five to primary seven, girls are more sexually advanced than boys, and have more needs for sexuality education*.

(senior man-rural)

*I meet girls 2–3 times in a term. I teach them to be clean especially during menstruation. Some girls are careless. A man is not supposed to see menstrual blood. I also teach them to respect elders but also to avoid men. I tell them the dangers of AIDS and accepting money from men*.

(senior woman rural)

### 3.2. Gender and Initiation of Sexual Relationships

The sexual relationships that prevail in Mbarara and Uganda depict boys as sexually active and the only beings sanctioned to initiate such relationships. The findings show an overwhelming opposition to girls initiating a love relationship. These are some of the responses from a focus group discussion with girls:

Interviewer: Who asks for love, a boy or girl?

Girl: Boy.

Interviewer: What happens when a girl asks a boy?

Girl: Can a girl ask a boy for love? I have never heard of that; that would be a prostitute. She will have ashamed us as girls because a girl never advances for sex, even our teacher told us about it in class (FGD Girls semi urban).

Interviewer: What would you think of your friend if she asks a boy for love?

Girl: I would think she is not normal (Girl, IDI rural).

Girl: I would break off that friendship. Her conduct would be regarded as strange (IDI, Girl urban).

Girl: *If I am a boy I know you want to kill me (IDI, Girl rural).*

It should however be noted that the norm appears to be changing with some girls arguably manifesting some level of agency. It was reported that some girls initiate sexual relationships—only that they typically use subtle but clear ways to convey their message. If a girl admired a boy, she could touch him or struggle to sit with him in class. Sometimes she can send a message by going through her friends or the friends of the boy. According to the boys, it is only brave girls that can advance, and such, are not common. The boys believe that the normal and right procedure is for a boy to ask a girl:

*God designed it that a boy should ask a girl and not vice-versa*.

(Boy FGD urban)

*Some girls can show you signs that they are interested, but it is not normal. You have to be wary of such girls who initiate sexual relationships. They could be having HIV*.

(Boy FGD urban)

The only means identified to commence a sexual relationship by boys was giving money or gifts to the girls. For some girls with no economic opportunities for themselves, entering into such relationships is the only way to obtain money to buy food, pads or books. By giving such gift, a boy or man can show his interest in a sexual relationship with a girl. If she does not want the relationship, the girl has to decline those gifts or else she has to account for them by giving in her body:

*If you want a girl, however rigid she might be, you give her money or a gift. Once she accepts it, you know you will win her over. You keep giving her in bits and not too much because if she knows you have a lot of money she will always come back for more*.

(IDI Boy Rural)

The poverty and economic dependency on the side of girls has led to such constructions that girls can be won over by money, and that boys have the capacity to measure what is just enough for the girls. However, it was also reported that some girls may get the money but still reject sexual activity with those that give them the money. In some instances, such can result into rape or defilement.

### 3.3. Division of Labour, Strength and Future Aspirations

Findings from this study uphold the widely documented view that girls and boys in Uganda still have different gender roles. The division of roles was stronger in rural areas than urban areas, contrary to the claim by government elites that affirmative action has evened up the gender question. In rural areas, girls reported doing typical traditional work for women like cooking, cleaning, collecting firewood, nursing and babysitting whereas boys go out to graze animals, milk cows, taking products to the market and sometimes fetching water. Findings indicate that whenever boys did not have sisters/girls at home, they performed girls’ duties of cooking and washing dishes. However, in the urban areas, while the divide still exists, it was not that substantial mainly because children (boys and girls) in towns have more to do with housework than outdoor activities. According to the boys and girls in the interviews, there are certain activities that need a lot of energy (strength) and therefore have to be done by boys. Thus, adolescents believe in the dominant stereotype that boys are stronger than girls and that outdoor activities are dangerous for girls.

*Boys are more energetic. I can carry only a 10 L jerican but for him he can ride with a full milk can of 20–25 L on the bicycle*.

(IDI girl urban)

*For us girls we do not go out to graze because it is dangerous for us*.

(IDI Girl Rural)

*Boys are lucky, sometimes you are in the house cooking. You serve them milk and food and for them they are just in the house seated. After the meal, you even clean up the dishes, and often get too exhausted to do your school assignments. You sometimes wish you were a boy who would sit, wait to be served, and have enough time for your school assignments*.

(IDI Girl semi-urban)

The gender role divide is reflected in future career aspirations of adolescents. Unlike pupils from the urban areas who have seen women doctors, almost all the girls in the rural areas desire to become nurses and primary school teachers. Some girls expressed with doubt if girls can become doctors:

Interviewer: What do you want to become in future?

Girl: A nurse.

Interviewer: Why? Can’t you become a doctor?

Girl: Most doctors are men (IDI Girl semi-urban).

Boy: I want to work in a garage, be a mechanic and get my own garage (boy semi-urban).

On the other hand, none of the boys mentioned nursing as their preferred profession, but only a handful in the rural area mentioned that they would like to become teachers. The majority of the boys aspire to become doctors, engineers, pilots and police men or soldiers. Because of their socially constructed gender roles (like baby-sitting, cooking), most girls mentioned marriage as an important achievement they want to accomplish. They had a desire to get more information concerning handling men and how to conduct themselves in marriage. While they dream to take on professional courses like teaching, they mainly want to be awarded respect by society if they have a successful marriage:

Interviewer: What kind of information would you like to hear that is never taught to you?

*They don’t tell us how to do things when we get married because we hear that before you get married, you should first visit your aunt to teach you some of these things*.

(FGD Girls Rural)

How to conduct yourself when you are going to get married and when you are married

(IDI Girl Rural)

*I would like to know how to handle marriage and pregnancy*.

(IDI Girl semi-urban)

### 3.4. Gender and Value Attached to Different Sexes

Our findings indicate that most girls loved being girls but hated the aspect of menstruation:

*“For me I hate being in my monthly periods, and I regret being a girl”*.

(Girl FGD semi-urban)

Given that menstruation of Ugandan girls typically arrives without prior preparation of the adolescents, it is difficult for the girls to psychologically accept it. Others however indicated that they were comfortable with menstruation because it is a sign of fertility. The ability to give birth was one of the most important things that girls loved and valued about themselves:

*For me I like experiencing periods. Much as it is painful, I have to endure because you cannot produce if you don’t get them and we would like to produce children*.

(FGD girls rural)

While some girls endure menstruation, it makes girls hate school because in the context of a poor country like Uganda, girls normally lack sanitary pads and soap to use.

*It’s not good being a girl because there are problems like menstruation. You feel a lot of pain and boys disturb us asking us very many questions in case they know you have started menstruating*.

(Girl semi-urban)

*I haven’t got my periods but I fear them, they can embarrass you at school*.

(IDI Girl 13 rural)

Boys pointed out that it is an honour to be a boy/man:

*“No one can ever wish to be a girl in life”*.

(Boy FGD rural)

While boys are aware that they have to work and become breadwinners for their families, they never wished to be girls because of the hardships associated with painful processes like menstruation and child birth:

*“At least wet dreams come and go. But menstrual periods keep coming”*.

(Boy FGD urban)

Moreover, they indicated that girls perform hard house chores and are never allowed to get out from home.

One of the challenged social norms in the study area however was the reduced segregation between boys and girls in terms of education, care and provisions. Boys complained that girls are favoured when it comes to shopping for clothes, edibles or other items for school. Girls seem to be getting more of what they ask for from parents than the boys. This is different from certain parts of Uganda where girl-child education is not given significance (girls may not continue with school or may be placed in poor schools as compared to boys when parents are faced with financial constraints). In this study, findings from boys, girls, teachers and parents, indicated that there was a more egalitarian tendency in regard to education access.

*A parent will send you to a bad school depending on your academic performance; if you have performed badly you go to a bad school. Not because you are a boy or girl* .

(IDI Girl urban)

*We don’t segregate our children (boys and girls) like our parents and grandparents did. We know that when a girl gets education she becomes an important person*.

(Male parent FGD Rural)

However, differences in values attached to a boy and girl child were persistent. Boys expressed that it was powerful to be a boy because they learnt from their parents that boys take over from their fathers when their fathers are away. And in case the father died, boys inherit property and look after the home rather than the girls:

*A woman’s role is to remain at home only*.

(Boy FGD Urban)

A girl also expressed the value attached to the boy child:

Girl: I want to have 2 children, a boy and a girl.

Interviewer: Supposing you get only girls?

Girl: *Ai bambe!!! (Meaning Uhhhhh); In our society when you produce girls only it’s like you have no children. You know that* (IDI Girl rural).

A parent stated:

*I have five girls. People say I have produced nothing but prostitutes. That I have no child because I have no boys. I feel bad that I have not yet given my husband a boy. When girls grow, they get married to other families. But a boy stays home and makes the family grow and continue. With girls, the family is no more. My husband is now sleeping with other women looking for a boy. He has already brought me one boy from another woman. I am looking after him. I am also still producing may be God will bless me with a boy. While I would be happy getting a boy, I still love my girls. And I want them to study. When they study, they can become important persons and help me*.

(Female parent semi-urban)

## 4. Discussion

The study aimed at understanding sexuality gender norms among young adolescents, parents and teachers and how they influence adolescents’ sexual dynamics. While Muhanguzi [3] and Iyer et al. [24] discuss the norms among older adolescents in central Uganda, this study explored whether these norms already manifest among the age of 12–14 years in western Uganda. Findings indicate that the agency of an individual actor is challenged by gender norms and stereotypes governing the attitudes and behaviour of adolescents. These gender norms are an important mediating factor in their sexual and reproductive experience [39]. Gendered norms are taught at home by parents. But as Bandura argues, learning and modelling takes place in different ways by different social referents which include extended family, teachers, peers and the media [7]. Various aunties play a complementary role by ensuring that girls become well-versed in the socially “appropriate” feminine behaviours and roles. These include the “proper” ways of how a good girl should sit, prepare food, conduct herself, respect elders and so on [40]. On the other hand, boys typically perform duties largely outside the home and are not very restricted in their movements. This finding is similar to that of Ngabaza [40] who found that boys and girls are socialised to perform different roles, have different expectations from parents, aunties and the wider communities. The differences exhibited in their aspirations like the values girls attach to motherhood, marriage, care giving and admiration for formal-sector jobs like nursing and teaching are a significant part of long-term gender ideals [14]. Such aspirations are both drivers to, and results of, unequal power relations [14], which are key aspects of sexuality.

The masculine behaviour was already present among boys aged 12–14. Boys engage with multiple partners, enjoy free movement, engage in economic activities and have the capacity to use the money to win over girls into sexual activity. By contrast, neither the boys nor the wider society expect the same behaviour from the girl-child. Girls are socially expected to be nice, submissive, and have restricted movements. The control of resources by men leads to un-equal power relations whereby girls/women cannot negotiate safer sex [4]. Unequal economic power restricts a girl’s sexual agency and could result in coercive sexual practices [3]. However, the norm or practice of multiple partners and polygamy for boys/men is contested by girls and women. Thus this is a practice that is done without girl’s knowledge, which can be considered as a sign of girl’s agency. While polygamy or multiple partners is legitimised by cultural norms, and thus providing the conduit through which agency is realised [19], girls/women continuously interact and live within these structures and participate in avenues to challenge them [19]. Other studies like Haberland [41] and De Meyer [39] reveal that more egalitarian gender attitudes are related to higher use of protection/contraceptives and easier communication about sex within the couple than those that had negative gender norms.

Results from this study indicate that female sexual activity is restricted and girls who express sexual agency are considered to be prostitutes or abnormal. Both boys and girls agreed that it was quite abnormal and strange if a girl proposed a sexual relationship to a boy. This is similar to what Muhanguzi [3] found in her study among older adolescents in Uganda. It should however be noted that girls have continuously expressed their sexual feelings though using subtle ways to convey their message. This has also been reported by Nyanzi [23] in her study among older adolescents in Uganda where girls are no longer expected to be amateurs but at the same time not lose their virginity. The stereotypical gender norm depicting boys as sexually active and girls as dormant with less (sexual) agency was also reflected in De Meyer et al. [39]. Our findings show that girl’s movement was more restricted compared to that of boys. Manifested in quotes like “*girls need to be locked up*” and gender stereotypes that girls naturally attract men, the dominant social norms give parents and teachers a mandate to control the girl-child more seriously than their male counterparts. Girls are socially expected to control their own sexuality but also take responsibility for boys’ sexualities because pregnancy is deemed to be girl’s responsibility. Moreover, as [37] reports, girls who get pregnant may get disowned by their families and have no guarantee that they will be accepted by the boy’s/man’s family. The notion of girls being punished for becoming pregnant was also found in Rwanda [22]. This is also reflected in Varga’s study where it was found that nothing ever happens to a boy if he does not accept paternity [42]. 

Moreover, while sexuality education should involve teaching of gender equality, to assist in changing patriarchal attitudes towards women/girls, our study suggests that sexuality education by teachers and parents gave preferentially higher attention to the girls compared to the boys. From this perspective, sexuality education mainly focused on biological aspects like hygiene, and control of girls’ sexuality. Girls were taught how to control themselves from male advances, respecting elders and helping with household chores. Tamale [31] shows that sexuality education taught in Uganda basically reinforces patriarchal control of female sexuality where girls are taught to be submissive to men. Teachers’ perceptions are shaped by gendered values which shape girls and boys differently [9]. A study done by Iyer and Aggleton, [43] (p. 6) in central Uganda found that teachers believed that boys need to behave like men and be in control while girls’ first responsibility is “…” having respect, and value for, their bodies… they are the “Mothers of Tomorrow”. Moreover, findings of this study also indicated that parents did not give girls an environment to express their worries regarding their sexuality. According to Svanemyr, et al. [15], gender norms that emphasize silence mostly for girls in obtaining information do not create a safe environment for their agency and wellbeing.

The legal system which would be supportive of addressing child protection and gender inequalities from local councils (courts), the police, and probation officers to Courts of Judicature is still weak (thanks to the high level of corruption in the Ugandan police force) and patriarchal. It is undermined by cultural values where sexuality is treated as a private matter [35]. This, coupled with poverty among the parents who may forego legal procedures when the culprits offer money [35] or choose to get their daughter married and save them the shame of keeping a pregnant daughter [33], has reinforced, not transformed, socially embedded patriarchal norms.

## 5. Conclusions

Our findings point to several potential target areas for programming to improve sexual and reproductive health among adolescents. One is that gender norms form early in life and create unequal power relations that constrain adolescents from exercising agency with regard to their sexuality and health. Thus, any program targeting behavioural change needs to begin early in the lives of young children. Second, the sexuality education that should address the unequal gender relations seems to be propagated by teachers and parents in Uganda. Thus, any program that aims at behavioural change without addressing the socio-cultural norms that perceive men as strong and all-knowing, and girls as passive and only designed for reproduction and men’s consumption will be counterproductive. A comprehensive gender-sensitive sexuality education focusing on values like equality, reciprocity, self-esteem and respect would be important. Such programs we argue will only be effective if they do not only target the teachers, but also parents and the community. This is because the primary agents of socialisation are families first, and then schools. Decisions taken by an individual are influenced by the socio-structure in which they live. Targeting schools with gender sensitive sexuality education, for example, without targeting the family would have diminutive impact. Third, in addition to educating children, teachers and parents about the values of gender equality, there is need for Uganda to strengthen the legal framework. An enabling legislative and policy framework is critically important in supporting adolescents, teachers and parents to address the health and wellbeing of adolescents.

### Limitations of the Study

The study focused on how social/gender norms constrain young adolescents’ agency regarding sexuality. While we may not authoritatively claim that the sample selected for this study is representative of the whole country, we can claim that results can be moveable to most parts of Uganda and other parts of Africa and Asia that have similar levels of economic development and similar patriarchal norms. 

Future research needs to explain the difference between the norms that adolescents practice (actual or perceived) and the norms expected of them by others/referents (injunctive). It would also be helpful to explain the extent to which parents and teachers influence and the extent to which peer pressure influences adolescents. More studies are needed to explain dynamics like the level of household income, the dynamics of accessing the legal system and the policy environment surrounding sexuality in Uganda.
